# Analysis of white-light imaging-based features predictive for determination of lesion depths of superficial flat esophageal squamous cell carcinoma: a retrospective multicenter study from China

**DOI:** 10.1186/s40001-023-01153-z

**Published:** 2023-06-09

**Authors:** Bin Wang, Yadong Feng, Jie Song, Jifeng Ma, Yan Liang, Mengjie Li, Xiaofen Wang, Cui-e Cheng, Ruihua Shi

**Affiliations:** 1 Department of Gastroenterology, Zhongda Hospital, School of Medicine, Southeast University, 87 Dingjiaqiao Road, Nanjing, 210009 China; 2 Department of Gastroenterology, the Affiliated Changshu Hospital of Nantong University, Changshu No.2 People’s Hospital, 18 Taishan Road, Suzhou, 215500 China; 3 Department of Gastroenterology, Maanshan 17th Metallurgy Hospital, 828 West Hunan Road, Maanshan, 243011 China

**Keywords:** Superficial esophageal squamous cell lesion, Endoscopic features, White light imaging, Invasion depth, Endoscopic diagnosis

## Abstract

**Objectives:**

Endoscopic diagnosis of invasion depth of superficial esophageal squamous cell carcinoma (SESCC) by white-light imaging (WLI) modality remains difficult. This study aims to clarify WLI-based features which are predictive for invasion depth of SESCC.

**Methods:**

A two-phase study was performed by enrolling 1288 patients with 1396 SESCC lesions. Endoscopic appearances, clinical characteristics and post-operative pathological outcomes were collected and reviewed. The association between lesion features and invasion depth were analyzed. A predictive nomogram was constructed for prediction of invasion depth.

**Results:**

Among 1396 lesions in derivation and validation cohort, 1139 (81.6%), 194 (13.9%) and 63 (4.5%) lesions were diagnosed as lesions confined into the intraepithelium or the lamina propria mucosa (T1a-EP/LPM), lesions invading the muscularis mucosa (T1a-MM) or superficial submucosa (T1b-SM1) and tumor with moderate invasion into the submucosa or deeper submucosal invasion (≥ T1b-SM2), respectively. Lesion length > 2 cm (*p* < 0.001), wider circumferential extension (*p* < 0.001, 0.002 and 0.048 for > 3/4, 1/2–3/4 and 1/4–1/2 circumferential extension, respectively), surface unevenness (*p* < 0.001 for both type 0-IIa/0-IIc lesions and mixed type lesions), spontaneous bleeding (*p* < 0.001), granularity (*p* < 0.001) and nodules (*p* < 0.001) were identified as significant factors predictive for lesion depth. A nomogram based on these factors was constructed and the values of area under the Receiver Operating Characteristics curve were 0.89 and 0.90 in the internal and external patient cohort.

**Conclusions:**

Our study provides six WLI-based morphological features predicting for lesion depth of SESCC. Our findings will make endoscopic evaluation of invasion depth for SESCC more convenient by assessing these profiles.

**Supplementary Information:**

The online version contains supplementary material available at 10.1186/s40001-023-01153-z.

## Introduction

It has been widely accepted that therapeutic strategy for superficial esophageal squamous cell carcinoma (SESCC) is based on careful and accurate preoperative endoscopic assessment. One main aim is to evaluate the invasion depth, which is correlated with risk of long-term lymph node metastasis [1]. Magnifying endoscopy combined with narrow band imaging (ME-NBI) or blue laser imaging (ME-BLI) has been widely used to access the invasion depth of SESCC preoperatively [1, 2], enabling a diagnostic accuracy of 90% by an expert endoscopist [3].

Despite of magnifying endoscopy systems have been introduced since many years [4], such systems are not popularly used in general practice. There are some reasons for this current status. First, interpretation of results from ME-NBI or ME-BLI is an experience and skill dependent procedure, as reflected by the truth that misdiagnosis from inexperienced endoscopists is high [5–9]. Second, there are not sufficient numbers of well-trained endoscopists who are competent for manipulation of magnifying endoscope systems. Third, ME-NBI or ME-BLI platform is not available in municipal and/or primary hospitals due to a high cost. Therefore, it will be much appreciated that lesion depth of SESCC can be determined by white-light imaging (WLI) solely.

In recent years, WLI-based assessment of lesion depth of SESCC has gained much interest in recent years [10–12]. However, such diagnosis remains challenging. Since diagnosis relevant imaging features have not been fully clarified, diagnostic accuracy, interobserver and intraobserver agreements are unsatisfactory. Therefore, to reveal diagnosis relevant features which can be predictive for invasion depth of SESCC is clinically significant. The main aim of this study was to explore those diagnostic relevant WLI-based endoscopic features for determination of lesion depth of SESCC.

## Materials and methods

### Study design

**Fig. 1:**
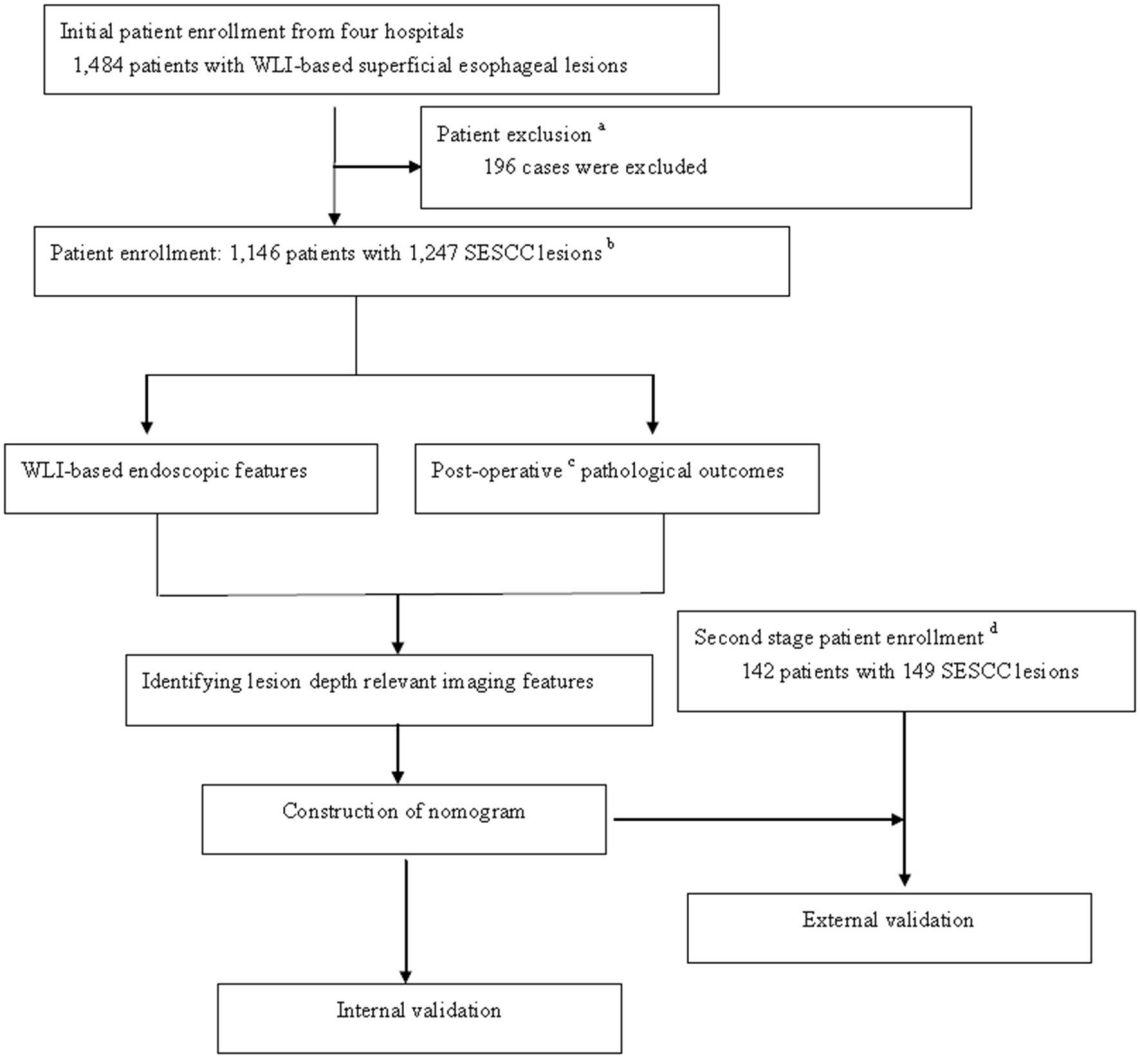
Flowchart of this study. a: 30, 23, 53, 55 and 35 patients were excluded due to inflammatory lesions, submucosal tumors, insufficient endoscopic or pathological records, protruding or excavated early ESCC, and advanced ESCC, respectively; b: enrolled patients were from four academic hospitals. Finally, 413, 307, 200 and 226 patients were enrolled from Zhongda Hospital Southeast University, First Affiliated Hospital of Nanjing Medical University, Huai’an First People’s Hospital affiliated to Nanjing Medical University and Taizhou People’s Hospital Affiliated to Nantong University; c: endoscopic resection or esophagectomy; d: 101 and 41 patients were recruited from Zhongda Hospital Southeast University and Taizhou People’s Hospital Affiliated to Nantong University, respectively. *WLI* white-light imaging, *SESCC* superficial esophageal squamous cell carcinoma

This was a two-stage study, which consisted of: (1) analyses of relevant key WLI features predictive for lesion depth of SESCC; (2) construction and validation of a nomogram for prediction of lesion depth of SESCC. In the first stage, image features predictive for lesion depths of SESCC was evaluated. In the second stage, a nomogram was constructed, and its efficiency was evaluated by an external patient cohort. The flowchart of this study is listed in Fig. 1. This study was registered on chineseclinicaltrials.gov (ChiCTR1900028524).

### Patient inclusion and exclusion criteria

The inclusion criteria of patient enrollment were: (1) SESCC; (2) underwent endoscopic resection or esophagectomy; (3) with clear pre-operative WLI images; (4) with complete pre-operative endoscopic results and post-operative pathological results. Exclusion criteria were: (1) protruding and/or excavated lesions; (2) inflammatory lesions, submucosal tumor, refluex esophagitis, low grade intraepithelial neoplasia, high grade intraepithelial neoplasia or advanced ESCC; (3) patients who did not undergo endoscopic resection or esophagectomy; (4) incomplete pre-operative endoscopic assessment and/or post-operative pathological results.

### Data collection and definition

Details of endoscopic appearances from each lesion, including lesion location, lesion length, circumferential extension, macroscopic appearance, along with surface characteristics (white coating, spontaneous bleeding, granularity and nodules), were retrospectively gathered and assessed by three experienced endoscopists independently. Post-operative pathological outcomes of invasion depth were also recorded.

**Fig. 2:**
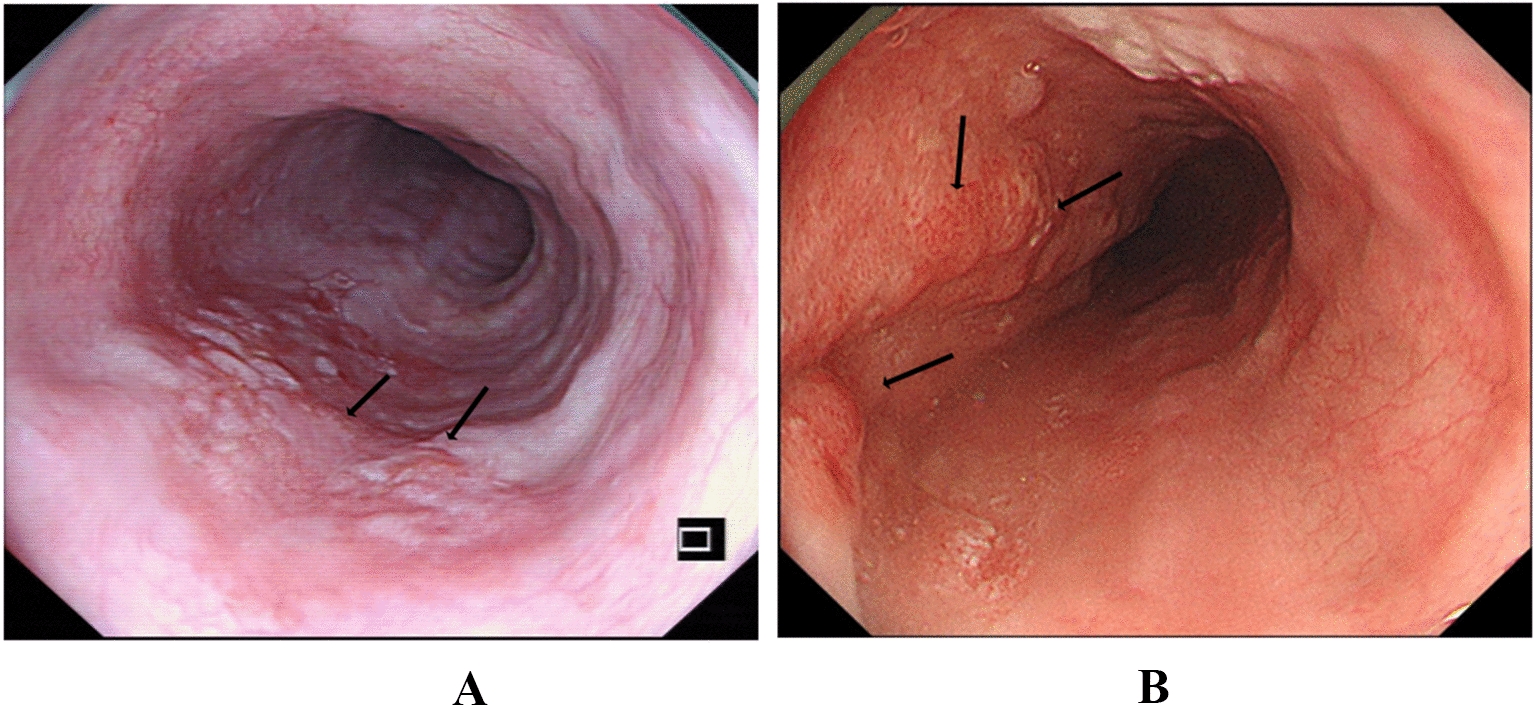
Typical WLI-based endoscopic appearance of granularity and nodularity of SESCC. WLI: white-light imaging; SESCC: superficial esophageal squamous cell carcinoma. A protrusion in size of < 5 mm or ≥ 5 mm was defined as granularity (**A**) and nodularity (**B**), respectively. Also, granular and nodular protrusions were shown by black arrows

Lesion location was identified according to the Japanese Classification of Esophageal Cancer [9]. The morphological features were defined according to the Paris classification [13]. Granularity and nodularity were defined as WLI-based gross protrusion in size of < 5 mm or ≥ 5 mm, respectively (Fig. 2). Lesion unevenness was defined as lesions in type 0-IIa, type 0-IIc or in a mixed type. The post-operative pathological results were evaluated by the diagnostic criteria of the Japanese Esophagus Society [9]. Lesion depth was classified into three categories [9]: lesions limited to the epithelium or lamina propria mucosa (T1a-EP/T1a-LPM); tumor invading the muscularis mucosa or superficial submucosa (T1a-MM/T1b-SM1); and tumor invading the submucosa or deep submucosal invasion (≥ T1b-SM2), respectively.

### Statistical analysis

Data were first checked for normality by Kolmogorov–Smirnov analysis, and were log-transferred if they did not meet the normality distribution. Continuous variables are presented as means ± standard deviation (SD) or medians and inter-quartile range (IQR), and categorical variables are expressed as frequencies or percentages. The Chi-square test, Wilcoxon test, Spearman’s correlation test, univariate and multivariate logistic regression analysis were performed using SPSS (SPSS v26.0 for Windows; SPSS Inc) and PyCharm (2020.2.1). A *p* value < 0.05 was considered statistically significant. The nomogram was constructed with the statistical software package R Version 4.0.5.

## Results

### Patient enrollment

In the first stage, 1484 patients who were admitted between January 2017 and November 2020, were initially enrolled. Among these, 30, 23, 53, 55 and 35 patients were excluded due to inflammatory lesions, submucosal tumors, insufficient endoscopic or pathological records, protruding or excavated early ESCC, and advanced ESCC, respectively. Finally, 413, 307, 200 and 226 patients were enrolled from Zhongda Hospital Southeast University, First Affiliated Hospital of Nanjing Medical University, Huai’an First People’s Hospital affiliated to Nanjing Medical University and Taizhou People’s Hospital Affiliated to Nantong University, respectively. These 1146 patients with 1247 SESCC lesions were used as derivation cohort. In the second phase, 101 patients with 104 SESCC lesions and 41 patients with 45 SESCC lesions, who were hospitalized at Zhongda Hospital Southeast University and Taizhou People’s Hospital Affiliated to Nantong University between February 2020 and May 2021, were recruited and set as the validation group.

### Endoscopic appearances, pathological outcomes and lesion distribution

Outcomes of endoscopic appearances and clinicopathological outcomes are listed in Table 1. In the derivation cohort, 109 (8.7%), 804(64.5%), 334 (26.8%) lesions were located at the upper, middle and lower thoracic esophagus, respectively. In addition, 50 (4.0%), 964 (77.3%), and 233 (18.7%) lesions were classified as type 0-IIa or 0-IIc, type 0-IIb and a mixed type, respectively. Among these 233 lesions, 28** (**2.2%), 69(5.5%), 82 (6.6%), 50(4.0%) and 4(0.3%) lesions were categorized into type 0-IIa + IIc, type 0-IIb + IIc, type 0-IIb + IIa, type 0-IIa + IIb, and type 0-IIc + IIb, respectively. Six hundred and fifty-one (52.2%),288(23.1%), 237(19.0%) and 71(5.7%) lesions were found with a < 1/4, 1/4–1/2, 1/2–3/4 and > 3/4 circumferential extension, respectively. Three hundred and twenty-five (26.1%), 38(3.0%), 347(27.8%) and 79(6.3%) lesions were found with white coat covering, spontaneous bleeding, granular change and nodular hyperplasia, respectively. As for lesion depth, 1010(81.0%), 178(14.3%) and 59(4.7%) lesions were diagnosed as T1a-EP/T1a-LPM, T1a-MM/T1b-SM1 and ≥ T1b-SM2, respectively.

**Table 1:** Baseline characteristics of the study population

Variables	Cohort	
	Derivation (*n* = 1146)	Validation (*n* = 142)
Gender, *n* (%)		
Male	771 (67.3)	104 (73.2)
Female	375 (32.7)	38(26.8)
Lesion numbers, n	1247	149
Age, years, mean(range)	66 (38–89)	67 (47–82)
Lesion location, n (%)		
Ut	109 (8.7)	6 (4.0)
Mt	804 (64.5)	125 (83.9)
Lt	334 (26.8)	18 (12.1)
Lesion length, cm, (mean ± SD)	2.4 ± 1.5	1.9 ± 1.3
> 2.0 cm, n (%)	464 (37.2)	40 (26.8)
≤ 2.0 cm, n (%)	783 (62.8)	109 (73.2)
Macroscopic type, n (%)		
0-IIb	964 (77.3)	116 (77.9)
0-IIa/0-IIc	50 (4.0)	5 (3.4)
Mixed type	233 (18.7)	28 (18.8)
0-IIa + IIc	28 (2.2)	5 (3.4)
0-IIb + IIc	69 (5.5)	7 (4.7)
0-IIb + IIa	82 (6.6)	11 (7.4)
0-IIa + IIb	50 (4.0)	3 (2.0)
0-IIc + IIb	4 (0.3)	2 (1.3)
Surface characteristic, n (%)		
White coating	325 (26.1)	46 (30.9)
Spontaneous bleeding	38 (3.0)	1 (0.7)
Granular change	347(27.8)	53(35.6)
Nodularity	79 (6.3)	10(6.7)
Circumferential extension, n (%)		
≤ 1/4	651 (52.2)	110 (73.8)
1/4–1/2	288 (23.1)	12 (8.1)
1/2–3/4	237 (19.0)	21 (14.1)
> 3/4	71 (5.7)	6 (4.0)
Depth of invasion, n (%)		
T1a-EP/T1a-LPM	1010 (81.0)	129 (86.6)
T1a-MM/T1b-SM1	178 (14.3)	16 (10.7)
≥ T1b-SM2	59 (4.7)	4 (2.7)

*Ut* Upper thoracic esophagus, from the sternal notch to the tracheal bifurcation, *Mt* Middle thoracic esophagus, the proximal half of the two equal portions between the tracheal bifurcation and the esophagogastric junction, *Lt* lower thoracic esophagus. *T1a-EP/LPM* lesions confined to the epithelium or amina propria, *T1a-MM/T1b-SM1* lesions invade to the muscularis mucosa or slight invasion into the submucosa, *T1b-SM2* lesions with deep invasion into the deep submucosa. *0-IIa* slightly elevated type, *0-IIb* flat type, *0-IIc* slightly depressed type

In the validation cohort, 6 (4.0%), 125 (84.0%) and 18 (12.0%) lesions were identified to be located at the upper, middle and lower thoracic esophagus, respectively. Five (3.4%), 116 (77.9%), and 28 (18.8%) lesions were categorized into type 0-IIa or 0-IIc, type 0-IIb, and a mixed type, respectively. In addition, five (3.4%), 7 (4.7%), 11(7.4%), 3(2.0%) and 2(1.3%) lesions were determined as type 0-IIa + IIc, type 0-IIb + IIc, type 0-IIb + IIa, type 0-IIa + IIb and type 0-IIc + IIb, respectively. One hundred and ten (73.8%), 12(8.1%), 21(14.1%) and 6(4.0%) lesions were found with a < 1/4, 1/4–1/2, 1/2–3/4 and > 3/4 circumferential extension, respectively. Forty-six (30.9%), one (0.7%), 53 (35.6%) and 10 (6.7%) lesions were identified with white coat covering, spontaneous bleeding, granular change and nodular hyperplasia, respectively. One hundred and twenty-nine (86.6%), 16 (10.7%) and 4 (2.7%) lesions were diagnosed with an invasion depth of T1a-EP/T1a-LPM, T1a-MM/T1b-SM1 and ≥ T1b-SM2, respectively.

**Fig. 3:**
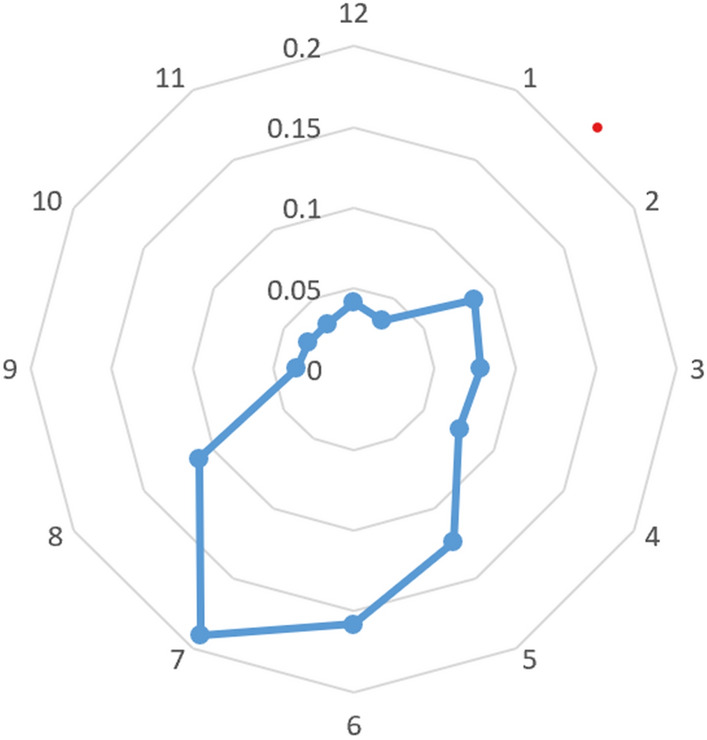
Endoscopic imaging-based distribution of SESCC lesions. About 58.3% and 36.4% of SESCC lesions were distributed at 5 o’clock to 8 o’clock and 2 o’clock to 5 o’clock in WLI images, respectively. SESCC: superficial esophageal squamous cell carcinoma

According to endoscopic outcomes from all participants, an area with high distribution of SESCC was identified. As shown in Fig. 3, 58.3% and 36.4% of SESCC lesions were distributed at 5 o’clock to 8 o’clock and 2 o’clock to 5 o’clock in WLI images, respectively. Relevantly, most majorities of SESCC lesions were found to be located at the left and posterior esophageal wall.

### Association of invasion depth with endoscopic appearances

Since lesion length is one key predictor to lesion depth of SESCC, thresholds for distinguishing different lesion depths were established by calculating Youden’s index [14]. In addition, 1.85 and 2.05 cm were set as the cutoff values for differentiation of T1a-EP/LPM, T1a-MM/T1b-SM1 and lesions deeper than T1b-SM2. Stratified lesion length by 2.0 cm, obvious morphological characteristics and surface features were present in large lesions (Additional file 1: Table S1).

The correlation between lesion characteristics and invasion depth is illustrated in Table 2. Among them, age (*p* = 0.015), lesion length > 2 cm (*p* < 0.001), wider circumferential extension (*p* < 0.001), uneven lesion surface (*p* < 0.001), spontaneous bleeding (*p* < 0.001), granular change (*p* < 0.001) and nodular hyperplasia (*p* < 0.001) were significantly correlated with invasion depth.

**Table 2:** Correlation between lesion characteristics and SESCC invasion depth in the derivation cohort

Variables	Invasion depth			*r*	*p* value
	T1a-EP/T1a-LPM	T1a-MM/T1b-SM1	≥ T1b-SM2		
	919 Patients	169 Patients	58 Patients		
	With 1010 lesions	With 178 lesions	With 59 lesions		
Gender, n, (%)				0.005	0.854
Male	620 (67.5)	117 (69.2)	38 (65.5)		
Female	299 (32.5)	52 (30.8)	20 (34.5)		
Age, years, mean(range)	65.5 (39–89)	66 (39–85)	69 (38–86)	0.069	0.015
Tumor location n, (%)				0.013	0.635
Ut	84 (8.3)	16 (9.0)	9 (15.3)		
Mt	663 (65.6)	108 (60.7)	33 (55.9)		
Lt	263 (26.0)	54 (30.3)	17 (28.8)		
Lesion length				0.220	< 0.001
> 2.0 cm, n (%)	325 (32.2)	100 (56.2)	39 (66.1)		
≤ 2.0 cm, n (%)	685 (67.8)	78 (43.8)	20 (33.9)		
Macroscopic type, n (%)				0.330	< 0.001
0-IIb	845 (83.7)	107 (60.1)	12 (20.3)		
0-IIa/0-IIc	32 (3.2)	11(6.2)	7 (11.9)		
Mixed type	133 (13.2)	60 (33.7)	40 (67.8)		
Surface characteristic, n (%)			25 (42.4)		
White coating	254 (25.1)	46 (25.8)	7 (11.9)	0.049	0.082
Spontaneous bleeding	20 (2.0)	11 (6.2)	25 (42.4)	0.133	< 0.001
Granular change	247 (24.5)	75 (42.1)	17 (28.8)	0.155	< 0.001
Nodularity	39 (3.9)	23 (12.9)		0.220	< 0.001
Circumferential extension, n(%)				0.470	< 0.001
≤ 1/4	578 (57.2)	58 (32.6)	15 (25.4)		
1/4–1/2	227 (22.5)	47 (26.4)	14 (23.7)		
1/2–3/4	163 (16.1)	56 (31.4)	18 (30.5)		
> 3/4	42 (4.2)	17 (9.6)	12 (20.4)		

*SESCC* superficial esophageal squamous cell carcinoma, *Ut* Upper thoracic esophagus, from the sternal notch to the tracheal bifurcation, *Mt* Middle thoracic esophagus, the proximal half of the two equal portions between the tracheal bifurcation and the esophagogastric junction; *Lt* lower thoracic esophagus. *T1a-EP/LPM* lesions confined to the epithelium or amina propria, *T1a-MM/T1b-SM1* lesions invade to the muscularis mucosa or slight invasion into the submucosa, *T1b-SM2* lesions with deep invasion into the deep submucosa. *0-IIb* flat type, *0-IIa* slightly elevated type, *0-IIc* slightly depressed type

Based on the triply classified lesion depths, those factors predictive to a deeper lesion depth of SESCC were assessed. According to univariate logistic analysis (Table 3), a deeper invasion depth was associated with older age (*p* = 0.018), lesion length > 2.0 cm (*p* < 0.001), wider circumferential extension (*p* < 0.001), uneven lesion surface (*p* < 0.001), spontaneous bleeding (*p* < 0.001), granularity (*p* < 0.001) and nodules (*p* < 0.001). Other variables, such as gender, lesion location and white coating covering, were not associated with invasion depth. Results from multivariate logistic analysis (Table 3) revealed that lesion length > 2.0 cm (*p* < 0.001), uneven surface (*p* < 0.001), wider circumference involvement (*p* < 0.001 for > 3/4 circumference involvement; *p* = 0.002 for 1/2–3/4 circumference involvement; *p* = 0.048 for 1/4–1/2 circumference involvement), spontaneous bleeding (*p* < 0.001), granular change (*p* < 0.001) and nodularity (*p* < 0.001) were predictive for a deeper invasion depth.

**Table 3:** Univariate and multivariate analyses for invasion depths of superficial esophageal squamous cell lesions

Variables	Univariate analysis		Multivariate analysis	
	OR (95%CI)	*p* value	OR (95%CI)	*p* value
Gender	1.001 (0.739–1.353)	0.999	NS	
Age	1.023 (1.004–1.042)	0.018	1.017 (0.996–1.039)	0.105
Lesion location			NS	
Ut	1.141 (0.684–1.902)	0.613		
Mt	0.789 (0.574–1.084)	0.144		
Lt	Ref	–		
Length > 2.0 cm	3.025 (2.266–4.039)	< 0.001	1.898 (1.343–2.686)	< 0.001
Macroscopic type			NS	
0-IIa/0-IIc	5.842 (4.255–8.020)	< 0.001	5.948 (3.168–11.167)	< 0.001
Mixed type	4.491 (2.477–8.142)	< 0.001	4.158 (2.965–5.824)	< 0.001
0-IIb	Ref	–	Ref	–
White coating	1.320 (0.969–1.799)	0.078		
Spontaneous bleeding	4.272 (2.289–7.980)	< 0.001	3.732 (1.927–7.236)	< 0.001
Granularity	2.234 (1.665–2.995)	< 0.001	1.548 (1.111–2.155)	< 0.001
Nodularity	5.529 (3.540–8.637)	< 0.001	2.542 (1.550–4.170)	< 0.001
Circumferential extension				
> 3/4	5.930 (3.530–10.115)	< 0.001	3.047 (1.692–5.490)	< 0.001
1/2–3/4	3.564 (2.474–5.135)	< 0.001	1.956 (1.275–2.998)	0.002
1/4–1/2	2.125 (1.465–3.083)	< 0.001	1.513 (1.003–2.282)	0.048
≤ 1/4	Ref	–	Ref	–

*Ut* Upper thoracic esophagus, from the sternal notch to the tracheal bifurcation, *Mt* Middle thoracic esophagus, the proximal half of the two equal portions between the tracheal bifurcation and the esophagogastric junction, *Lt* lower thoracic esophagus. *0-IIa* slightly elevated type, *0-IIb* flat type, *0-IIc* slightly depressed type, *NS* non-sense, and this analysis was not performed

### Generation and validation of the prediction model

**Fig. 4:**
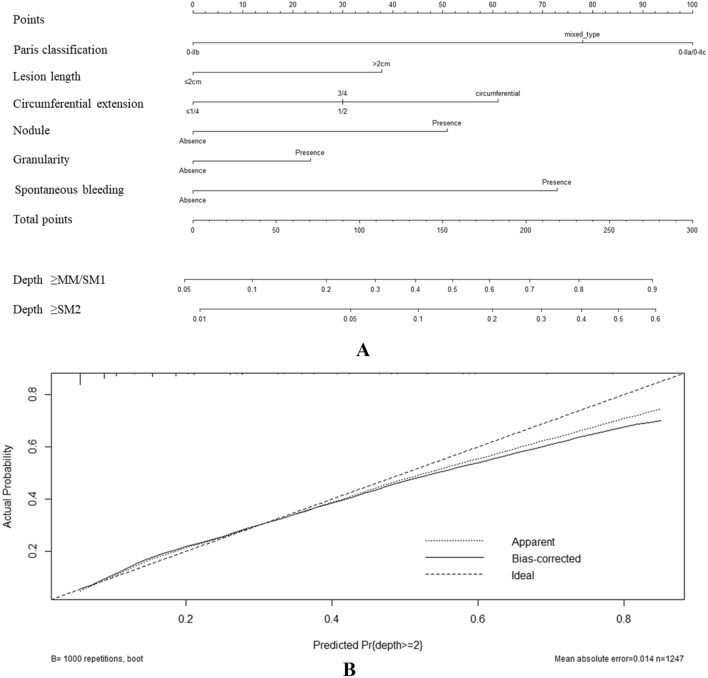
Nomogram predicting lesion depths of SESCC. This model (**A**) was constructed based on six diagnosis relevant WLI-based features, including Paris classification, lesion length, circumferential extension, nodularity, granularity and spontaneous bleeding. As shown in **B**, good agreement between prediction and observation was demonstrated. *SESCC* superficial esophageal squamous cell carcinoma, *MM/SM1* tumor invading the muscularis mucosa or superficial submucosa, *SM2* tumor invading the submucosa or deep submucosal invasion

**Fig. 5:**
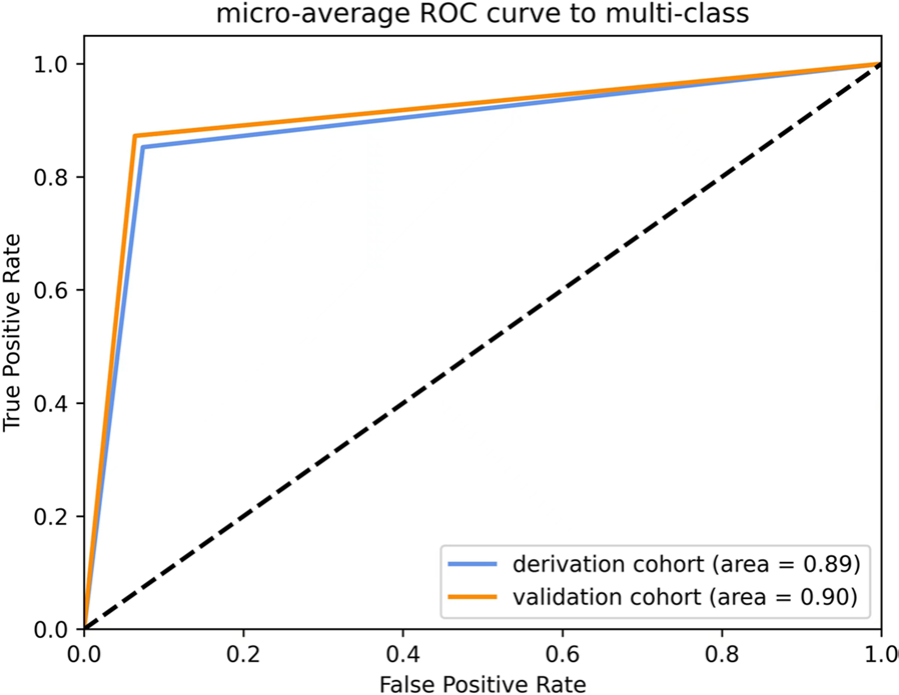
Prediction role of lesion depth of SESCC by this nomogram. As reflected in the Receiver Operating Characteristics (ROC) curves, the values of area under the ROC were 0.89 and 0.90 in the derivation and external cohort, respectively. *SESCC* superficial esophageal squamous cell carcinoma

A nomogram was constructed for predicting a deeper lesion of SESCC using identified risk factors (Fig. 4A). The calibration curve showed good agreement between prediction and observation in the derivation cohort (Fig. 4B). The area under the ROC curve (AUC) was 0.89 in the derivation cohort (Fig. 5). Characteristics of external patient cohort were de-identified and listed as Additional file 2: Table S2. Based on the AUC of 0.90, this model also showed a good performance in this external validation cohort (Fig. 5).

An example was shown for full understanding of the diagnostic performance of this nomogram. The total points of a type 0-IIb, lesion length < 2 cm, < 1/4 circumference involvement, granular, non-nodular and non-spontaneous bleeding SESCC was 26. The probabilities were 0.1 and less than 0.05, respectively, for lesion depth deeper than T1a-MM/T1b-SM1 and T1b-SM2, respectively. Relevantly, the predicted lesion depth of this lesion was T1a-EP/LPM.

## Discussion

In this multicenter study, six key morphological features relevant to WLI-based diagnosis of lesion depth of SESCC were identified. A nomogram, which yielded novel diagnostic outcomes, was constructed. Our findings provide a novel comprehension for pre-operative endoscopic assessment of SESCC lesion depth.

Currently, early detection of ESCC in China has been prompted by wide application of high definition WLI endoscopy platforms. However, endoscopic evaluation of lesion depth of SESCC under WLI is challenging [15–18], as reflected by limited performances by few expert endoscopists [12, 18]. Since diagnoses relevant morphological characteristics are not well-established, high interobserver and intraobserver agreements are hard to be achieved. In addition, diagnostic accuracy is not high, especially in those inexperienced endoscopists [12, 15–18]. Therefore, we aimed to figure out those significant morphological features under WLI.

In recent years, roles of endoscopic imaging-based morphological features for prediction of lesion depth of SESCC have been reported. In the study by Huh CW et al [19], ESCC lesions in protruding (type I) or excavated (type III) endoscopic appearance were with submucosal invasion [13]. Bae J et al[. 18] also reported that type I or III lesion shape was correlated with a deeper invasion. Their studies showed that some other surface changes, such as nodularity and granularity, were also associated with a deeper invasion depth. Since SESCC is always presented in a flat shape, previous studies [12, 18, 19] were biased by enrolling protruding and depressed lesions for analyses. In addition, sample sizes and case numbers of flat (type 0-II) ESCC lesions in these studies were limited. By contrast, we only focused on flat ESCC lesions in this study. Our findings revealed that surface unevenness, nodularity and granularity were associated with a deeper lesion infiltration. Similarly, previous studies [20, 21] also demonstrated that SESCC can be tentatively classified into clinical subgroups with different lesion invasion according to WLI-based lesion shape and surface features. In this study, spontaneous bleeding was first identified as another predictor for lesion depth, which may due to excessive submucosal angiogenesis in a relative advanced stage of disease. Based on our data, since most majorities of SESCC lesions were in a type-0 IIb shape, it is essential to perform co-analysis for gross lesion types and other surface characters.

Although endoscopic resection can be used as curable therapeutic method for large SESCC [21], the association between lesion size and tumor infiltration should be considered [21–23]. Lesion size is an independent factor for assessment of infiltration depth of large SESCC [24], and pre-operative endoscopic assessment for larger size SESCC lesions is prone to be under diagnosed. According to previous studies [21–23], cutoff value of SESCC lesion length for differentiation of lesion depth is not uniquely defined. These discrepancies are mainly due to different patient enrollment. In this study, a lesion length at 2.0 cm was established as the cutoff value, and a binary classification of lesion length was adopted for further analysis. Similarly, a wider circumferential extension was also found to be predictive for a deeper invasion depth, which was consistent with previous reports [22, 24]. All these indicate that pre-operative assessment should be performed for the aim of decreasing underdiagnosis rate of lesion depth in SESCC lesions in a large lesion size [24].

Ebi M et al. reported their experience of WLI-based diagnosis for identifying SESCC beyond T1a-MM invasion [12]. Bae J et al. showed a predictive nomogram for distinguishing SESCCs within upper submucosal invasion from those with a deeper invasion [18]. Clinical significances of these studies were limited due to insufficient classification of lesion depths of SESCC, which are recommended by the criteria of the Japanese Esophagus Society [9]. Taking advantages of precise classification of lesion depths of SESCC, our data provided a novel comprehension of lesion depth diagnosis relevant imaging characteristics in type 0-II SESCC. Subsequently, a nomogram model was constructed based on diagnostically significant factors, and a satisfactory predicting efficacy for lesion depth was demonstrated by an external cohort for validation. Herein, a mathematical model for pre-operative assessment of tumor invasion of SESCC was available.

Based on our data, majority SESCC lesions were located at the middle thoracic esophagus, followed by the lower thoracic part, which is consistent with that of advanced ESCC [25]. Our study also demonstrated that SESCC had a high spatial distribution zone. Therefore, it is worthwhile to examine the lower hemisphere of the middle part of thoracic esophagus carefully, which was considered to be with a high misdiagnosis of early ESCC [11].

The most interest of this study is that several key predictors have been established. Our findings may make endoscopic assessment of lesion depth of flat SESCC a simple, convenient and medical source saving procedure. Those lesions really need to be evaluated by magnifying endoscopy examination can be identified by assessing relevant features. In addition, our results will potentially contribute to improvement in diagnostic efficiency. There are some limitations of this study. First, due to the nature of a retrospective study, inter- and intra-observer disagreement may occur when evaluating detailed endoscopic appearances. However, to overcome this insufficiency, we defined unique criteria for evaluation of relevant morphological features. Second, this is a single-arm observational study using imaging from WLI modality only, and results from magnifying endoscopy were not set as control. Third, we only used pathological outcomes of SESCC as gold standard for analysis. Hence, SESCC lesions were classified into pathologically confirmed subgroups.

Taken together, our research will expand current knowledgement of WLI-based endoscopic evaluation of SESCC. In addition, our data are potentially useful for pre-operative endoscopic assessment of tumor invasion in SESCC.

## Supplementary Information

**Additional file 1: Table S1**. Clinicopathologic characteristics of all lesions stratified by lesion length.

**Additional file 2: Table S2**. De-identified clinicopathlogical results in patient cohort for external validation.

## Data Availability

All data and materials supporting the study have been included in the manuscript. All data are available upon request to the corresponding author for non-commercial purposes.
