# Supplementary material for: Analysis of white-light imaging-based features predictive for determination of lesion depths of superficial flat esophageal squamous cell carcinoma: a retrospective multicenter study from China
Source: Eur J Med Res. 2023 Jun 9;28:187. doi: 10.1186/s40001-023-01153-z (PMC10251637; doi:10.1186/s40001-023-01153-z)
Supplement: Supplementary file 1 — Additional file 1: Table S1. Clinicopathologic characteristics of all lesions stratified by lesion length. [file 40001_2023_1153_MOESM1_ESM.docx]

Table S1. The clinicopathologic characteristics of all lesions stratified by lesion length

| Variables | Lesion length | | | | |  | | | χ2 | | *p* value |
| --- | --- | --- | --- | --- | --- | --- | --- | --- | --- | --- | --- |
|  | ≤2.0cm | | | | >2.0cm | | |  |  | |  |
|  | 709 patients with 783 lesions | | | | 437 patients with 464 lesions | | |  |  | |  |
| Gender n, (%)  Male  Female | 478(67.4)  231(32.6) | |  | 297(68.0)  140(32.0) | | |  | | | 0.037 | 0.848 |
| Age, years, mean,(range) | 65(39-86) | |  | 66(38-89) | | |  | | | -1.544 | 0.122 |
| Tumor location n, (%)  Ut  Mt  Lt | 76(9.7)  511(65.3)  196(25.0) | |  | 33(7.1)  293(63.2)  138(29.7) | |  | | | | 4.858 | 0.088 |
| Macroscopic type, n (%)  0-IIb  0-IIa/0-IIc  mixed type | 635(81.1)  38(4.9)  110(14.0) | |  | 329(70.9)  11(2.4)  124(26.7) | |  | | | | 33.431 | <0.001 |
| Surface characteristics, n (%)  white coating  spontaneous bleeding  granular change  nodule | 184(23.5)  22(2.8)  178(22.7)  28(3.6) | |  | 141(30.4)  16(3.4)  169(36.4)  51(11.0) | |  | | | | 7.174  0.402  27.186  29.999 | 0.007  0.526  <0.001  <0.001 |
| Circumferential extension, n (%)  ≤1/4  1/4-1/2  2/4-3/4  >3/4 | 535(68.3)  165(21.1)  72(9.2)  11(1.4) | |  | 116(25.0)  123 (26.5)  165(35.6)  60(12.9) | |  | | | | -16.598 | <0.001 |
| Depth of invasion, n (%)  T1a-EP/T1a-LPM  T1a-MM/T1b-SM1  ≥T1b-SM2 | 685(87.5  78(10.0)  20(2.6) |  | | 325(70.0)  100(21.6)  39(8.4) | |  | | | | -7.677 | <0.001 |

Ut: Upper thoracic esophagus, from the sternal notch to the tracheal bifurcation; Mt: Middle thoracic esophagus, the proximal half of the two equal portions between the tracheal bifurcation and the esophagogastric junction; Lt: lower thoracic esophagus. T1a-EP/LPM: lesions confined to the epithelium or amina propria; T1a-MM/T1b-SM1: lesions invade to the muscularis mucosa or slight invasion into the submucosa; T1b-SM2: lesions with deep invasion into the deep submucosa. 0-IIb: flat type; 0-IIa: slightly elevated type; 0-IIc: slightly depressed type.
