# Supplementary material for: Analysis of white-light imaging-based features predictive for determination of lesion depths of superficial flat esophageal squamous cell carcinoma: a retrospective multicenter study from China
Source: Eur J Med Res. 2023 Jun 9;28:187. doi: 10.1186/s40001-023-01153-z (PMC10251637; doi:10.1186/s40001-023-01153-z)
Supplement: Supplementary file 2 — Additional file 2: Table S2. De-identified clinicopathlogical results in patient cohort for external validation. [file 40001_2023_1153_MOESM2_ESM.docx]

Table S2. De-identified clinicopathlogical results in patient cohort for external validation

| Variables | | Invasion depth | | |
| --- | --- | --- | --- | --- |
|  |  | T1a-EP/T1a-LPM T1a-MM/T1b-SM1 ≥T1b-SM2 | | |
|  | | 124 patients 14 patients 4 patients | | |
|  | | with 129 lesions with 16 lesions with 4 lesions | | |
| Gender, n (%)  Male  Female | | 90(72.6)  34(27.4) | 10(71.4)  4(28.6) | 0(0.0)  4(100.0) |
| Age, years, mean(range) | | 67(47-82) | 67(59-77) | 67(66-74) |
| Lesion location, n (%)  Ut  Mt  Lt | | 4(3.1)  109(84.5)  16(12.4) | 1(6.3)  14(87.5)  1(6.3) | 1(25.0)  2(50.0)  1(25.0) |
| Lesion length, cm,(mean ±SD)  > 2.0cm, n (%)  ≤ 2.0cm, n (%) | | 2.0±1.3  30(23.3)  99(76.7) | 1.9±1.3  6(37.5)  10(62.5) | 2.7±2.4  4(100.0)  0(0.0) |
| Macroscopic type, n (%)  0-IIb  0-IIa/0-IIc  mixed type | 105(81.4)  6(4.7)  18(14.0) | | 11(68.8)  0(0.0)  5(31.2) | 0(0.0)  0(0.0)  4(100.0) |
| Surface characteristic, n (%)  white coating  spontaneous bleeding  granular change  nodularity | 35(27.1)  1(0.8)  48(37.2)  6(4.7) | | 8(50.0)  0(0.0)  3(18.8)  2(12.5) | 3(75.0)  0(0.0)  2(50.0)  2(50.0) |
| Circumferential extension, n (%)  ≤1/4  1/4-1/2  1/2-3/4  >3/4 | 100(77.5)  9(7.0)  18(14.0)  2(1.6) | | 9(56.3)  3(18.8)  2(12.5)  2(12.5) | 1(25.0)  0(0.0)  1(25.0)  2(50.0) |

Ut: Upper thoracic esophagus, from the sternal notch to the tracheal bifurcation; Mt: Middle thoracic esophagus, the proximal half of the two equal portions between the tracheal bifurcation and the esophagogastric junction; Lt: lower thoracic esophagus. T1a-EP/LPM: lesions confined to the epithelium or amina propria; T1a-MM/T1b-SM1: lesions invade to the muscularis mucosa or slight invasion into the submucosa; T1b-SM2: lesions with deep invasion into the deep submucosa. 0-IIa: slightly elevated type; 0-IIb: flat type; 0-IIc: slightly depressed type.
